# Immunity against *Mycobacterium tuberculosis* and the risk of biologic anti-TNF-α reagents

**DOI:** 10.1186/1546-0096-12-45

**Published:** 2014-10-02

**Authors:** Kozo Yasui

**Affiliations:** Department of Pediatrics, Hiroshima-City Hospital, Moto-Machi 7-33, Naka-Ku Hiroshima, 730-8518 Japan; Department of Pediatrics, Okayama University Graduate School of Medicine, Dentistry and Pharmaceutical Sciences, Okayama, Japan

**Keywords:** Tumor necrosis factor-α (TNF-α), Tuberculosis (TB), Rheumatoid arthritis (RA), Juvenile idiopathic arthritis (JIA), Inflammatory bowel disease (IBD), Granulomatous infection, Latency, Dissemination

## Abstract

A third of the world’s population is exposed to *Mycobacterium tuberculosis* in their lifetime. Over eight million people develop a tuberculosis (TB) illness and 1.3 million people die from the disease every year. Acquired immunity (cytotoxic CD8+ T cells (CBT), Th1 CD4+ helper T cells) macrophages, and dendritic cells all play important roles in TB infection. Recently, it is well established that innate immunity as well plays a definitive role in the development of TB immunity under the effects of several cytokines, microbicidal proteins and Toll-like receptors. Meanwhile, the introduction and widespread use of biological disease-modifying anti-rheumatic reagents over the last 15 years worldwide has dramatically advanced and improved the standard care and prognosis of patients with rheumatoid arthritis (RA) and juvenile idiopathic arthritis (JIA).

However, as clinical experience with these drugs has grown, the risk of granulomatous infections, especially disseminated TB and fungal infections, has become apparent, especially because having RA or JIA may innately increase the risk of infection (bacterial, viral and fungal). The knowledge of basic immunology has also advanced over the past 10 years and adult and pediatric rheumatologists should increase their understanding of this dynamic between arthritis diseases, anti-TNF- α medications, and TB. This review will provide an up-to-date discussion of both the immunology of the TB organism in the human host and the pathophysiologic mechanisms of the TNF-α blockers in the development of secondary (disseminated) tuberculosis.

## Background

Biological reagents (so-called “biologics”) target cytokines and cell surface proteins. The use of these biological reagents has brought impressive and revolutionary improvements in clinical outcomes in the medical care of many rheumatic diseases, especially rheumatoid arthritis (RA) and juvenile idiopathic arthritis (JIA) as well as inflammatory bowel disease. Last year (2013) marks the tenth anniversary of the approval of the first tumor necrosis factor (TNF)-α antagonist for the treatment of RA in Japan. Clinical side effects have been tracked in Japan and other countries since the biologics were authorized and marketed as anti-rheumatic and/or immune-modulatory drugs.

The remarkably high incidence of tuberculosis (TB) in patients treated with TNF-α antagonists raises the intriguing question of the physiological role of TNF-α in the immune response [1–6]. There are several cytokines involved in the pathogenesis of RA including TNF-α. As the pathological role of TNF-α has become better understood, the physiological role of TNF-α in maintaining the latency of TB in granulomas in infected people has also been better clarified [4–6].

In the present manuscript, the articles about immunity against TB infections are reviewed and the information that should be kept in mind to ensure the suitable and safe use of biologics is described.

## Review

### Tuberculosis

**Figure 1:**
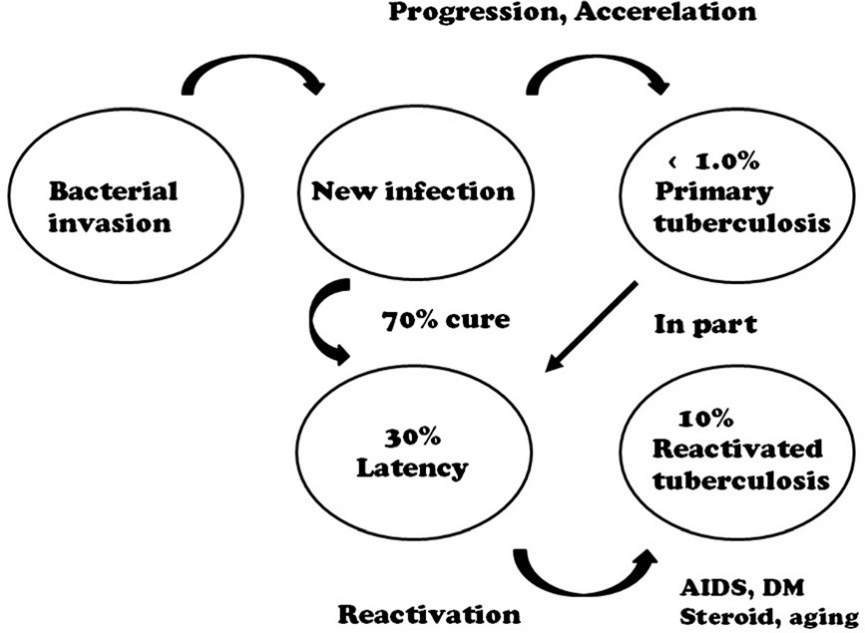
**Progression of** ***M. tuberculosis*** **infection.** The relationship between primary tuberculosis, latency, and reactivated tuberculosis and the possibility of the onset of disease. Reactivation is triggered by AIDS, DM, steroids, anti-TNF blockers, aging, and possibly other unknown factors.

A third of the world’s population is exposed to *Mycobacterium tuberculosis*. A clinical TB illness developed in 8.6 million people in 2012 and according to the statistics of the World Health Organization, 1.3 million people die from the disease every year worldwide [7]. Secondary TB, which follows primary TB, develops after the incubation period (latent infection). Re-activated TB disease often leads to secondary dissemination. Primary TB with the Ghon’s complex as well as hilar and cervical lymph node TB and pleurisy develops in approximately 0.5-1.0% of infected people, and the remaining are in a state of latent infection (latency) (Figure 1) [4, 8]. People infected with TB bacilli are usually asymptomatic during latent infection, and secondary TB develops in 10% of those by endogenous reactivation. Each granuloma contains viable TB bacilli; most likely, throughout the lifetime of the host, the maintenance of the granuloma is a prerequisite to allowing continued protection from the bacilli [2, 4, 8]. Factors disrupting the fine-tuned balance between mycobacteria and the maintenance of the granuloma will inevitably raise the risk of reactivation of the disease.

**Figure 2:**
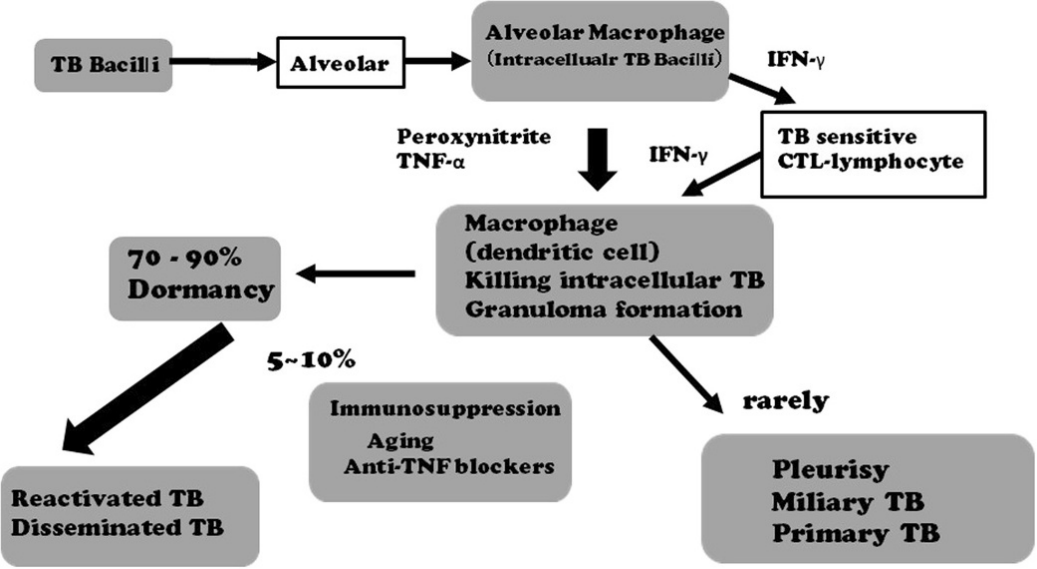
**Invasion of mycobacterium bacilli and the process of the onset of disease.** The relationship between cytokines and immunoregulatory cells. Abbreviations: TB, tuberculosis; IFN, interferon; TNF, tumor necrosis factor; CTL, cytotoxic T lymphocytes.

Most cases of adult disseminated TB are secondary tuberculosis induced by endogenous reactivation. Failure of the host immune mechanism (due to HIV infection, dosage of immunosuppressant medication, malnutrition, aging, and perhaps other unknown factors) could bring the onset of secondary TB. Certainly, the use of anti-TNF-α antibody drugs specifically may be a trigger (Figure 2).

### Immune response to tb and the mechanism of sterization of the tb bacilli

The understanding of the immune responses to TB bacilli has advanced during the past several years. Our present understanding is as follows:

#### Alveolar macrophages and epithelium cells

The bacilli are often ingested by alveolar macrophages after invading the lung tissue. The TB bacilli may also invade human alveolar epithelium cells through the action of mammalian cell entry protein on the cell surface [9, 10].

#### Induction of reactive nitrogen intermediates

In mice, the combination of the T-cell derived cytokine interferon-γ (IFN-γ) and TNF-α first induces antimicrobial activity in macrophages via the induction of reactive nitrogen intermediates (peroxynitrite) and killing peptides (defensin, cathelicidin) [11–13]. Superoxide anion production in phagocytes can sterilize the bacteria; however, these anions are not strong enough to kill tuberculosis bacilli because of the strong catalase activity and superoxide dismutase (SOD) action of the bacilli, through which the bacilli directly scavenge oxygen radicals [14, 15].

#### TNF-α activity

**Figure 3:**
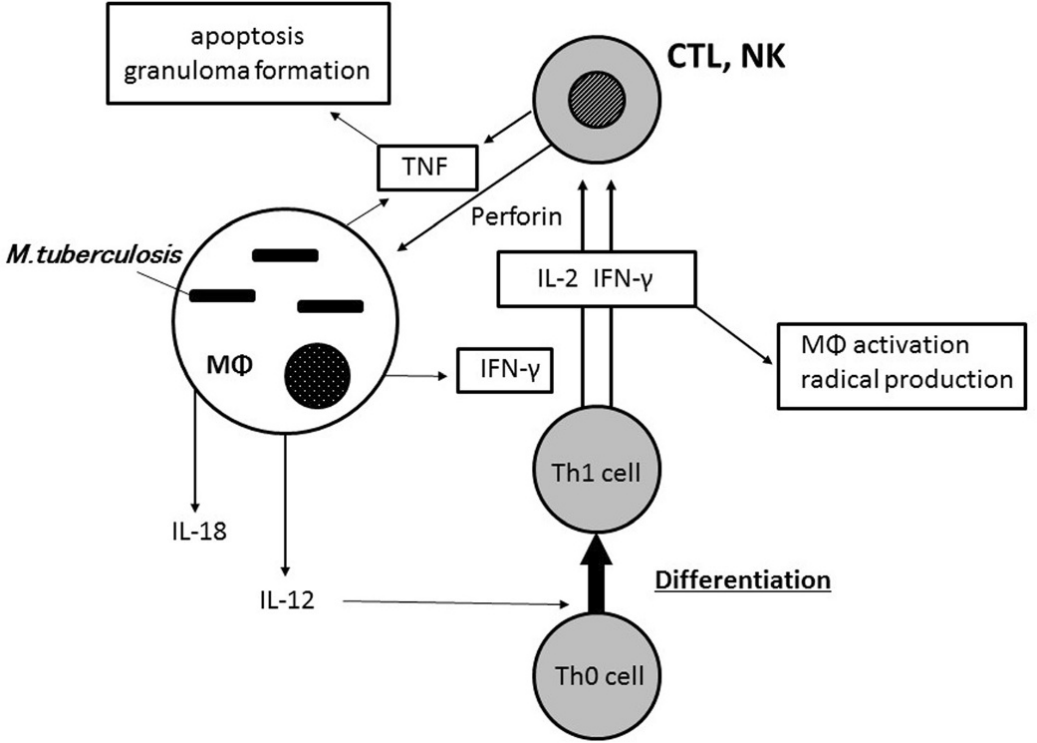
**Innate immunity and adaptive immunity in tuberculosis.** Abbreviations: MΦ, macrophage; IFN, interferon; TNF, tumor necrosis factor; CTL, cytotoxic T lymphocytes; IL, interleukin; Th, T helper.

IFN-γ and TNF-α are mainly produced from dendritic cells (DCs) and the activated macrophages [16, 17]. One of the major functions of TNF-α is the recruitment of monocytes and circulating antigen-specific T lymphocytes to the site of TB infection. TNF-α directs leukocyte movement, including its action on the vascular endothelium (intracellular adhesion molecule) and on the establishment of chemokine gradients [16–18]. TNF-α additionally activates CD8+ cytotoxic T cells (CTLs) that may be important because these cells release granulysin and directly kill intracellular bacteria [19]. TNF-α also promotes the maturation of monocytes to dendritic cells (DCs) and/or macrophages, inducing the antigen presentation of intracellular mycobacteria. TNF-α produced in a local infection site allows macrophages, natural killer (NK) cells and γδ T cells gather at the infection site and bring their activation. The activated CTL cells have the ability to produce perforin protein and TNF-α by itself, which guide TB-infected monocytes to apoptosis, which involves intracellular living TB bacilli, and to induce the autophagy of infected cells via activated macrophages [20] (Figure 3).

#### The role of dendritic cells

DCs are present in the epithelial layers, including the alveolar spaces in the lung, where they create a tight surveillance network [21, 22]. DCs have a special role in initiating T cell responses in TB infection. Local alveolar macrophages are poor antigen-presenting cells prior to their differentiation into DCs [23, 24]. This localization suggests that DCs present a first line of defense against inhaled foreign particles, including TB bacilli, in the process of antigen presentation [18].

#### Granuloma formation

**Figure 4:**
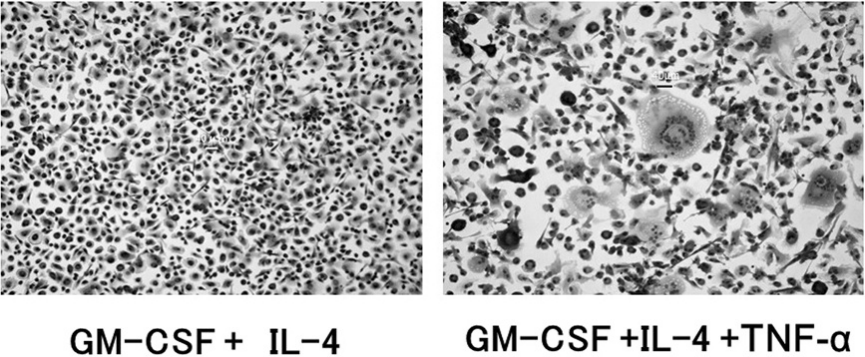
**The experimental cell cultures with several cytokines.** The characterization of peripheral blood monocytes that are cultured in GM-CSF (20 ng/mL) and IL-4 (20 ng/mL) in the presence or absence of TNF-α (20 ng/mL) are demonstrated. Representative images of cultured May-Grünwald-Giemsa-stained monocyte-derived cells (10x) are shown and Langhans-like cells are observed in the presence of TNF-α GM-CSF, granulocyte/macrophage colony-stimulating factor. Abbreviations: IL-4, interleukin-4; TNF, tumor necrosis factor.

**Figure 5:**
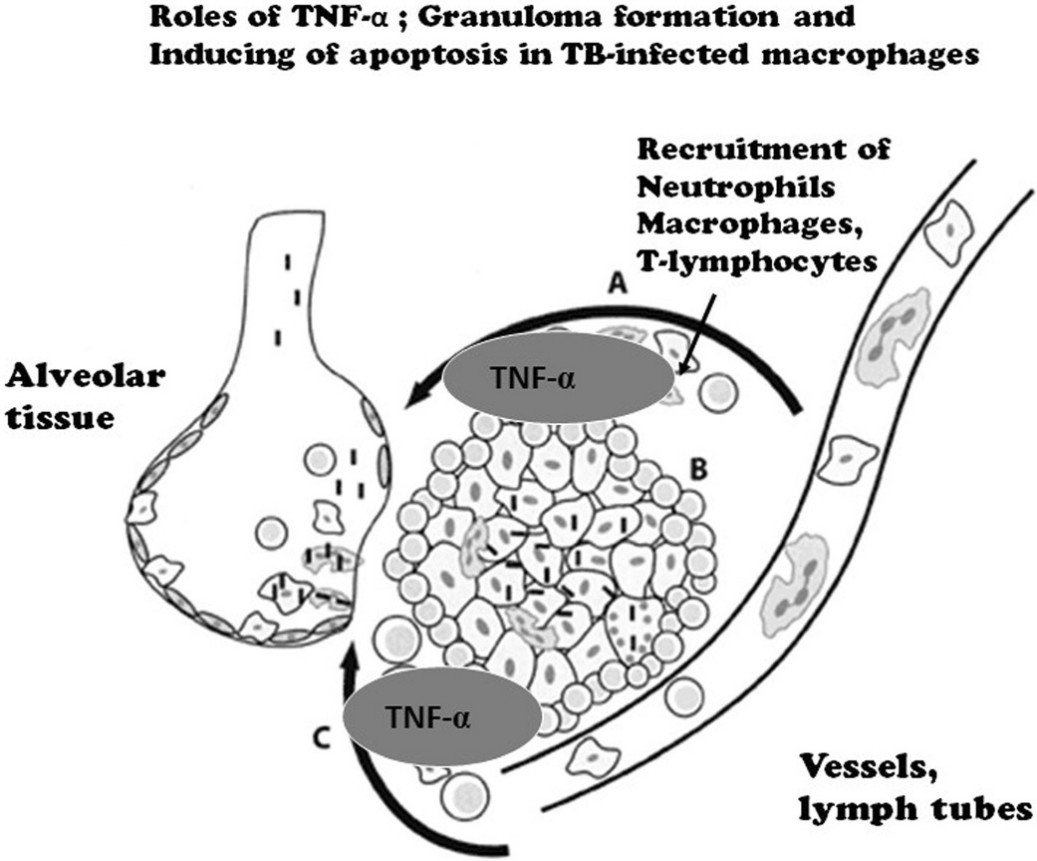
**Key functions of the tumor necrosis factor (TNF)-α cytokine in tuberculosis.** (A) TNF-α recruits neutrophils, macrophages and T-lymphocytes to the infection site; (B) The cytokine maintains granuloma formation in tuberculosis; (C) The autocrinal TNF-α is also produced and induces apoptosis of TB-infected cells.

Granuloma formation can prevent infectious expansion (dissemination). Langhans giant cells, lymphocytes, fibroblast cells surround the ingested TB bacteria [3, 18, 25] and in the presence of TNF-α, a granuloma is then effectively formed (Figures 4 and 5). The TB bacillus is captured in a granuloma but can survive inside [26]. The space is replaced by the caseation organization, and in most cases, the TB bacillus remains in a dormant state (latent infection). Caseation organization is exaggerated in the presence of TNF-α.

### Risk of biologics and tuberculosis

Rheumatic disease itself is associated with an increased risk for TB without the use of anti-TNF-α medications (approximately 2–4 times [27]). Seong and others have reported that the TB onset risk ratio in a population with rheumatic disease treated with infliximab in South Korea rose from 8.9 fold (95% CI, 4.6-17.2) to 30.1 fold [28]. Keane and colleagues published results based on the FDA Adverse Event Reporting System. They found 70 cases of TB in 147,000 patients who had received the treatment with infliximab, a TNF-α neutralizer [1]. The estimated rate of TB among patients with rheumatoid arthritis in United States is 24.4 cases per 100,000.

The infliximab-associated cases had unusual characteristics that may have caused the TB infection to be especially severe; 40 out of 70 cases presented extrapulmonary disease (17 of which were disseminated). The median interval between starting infliximab and the development of infection was only 12 weeks; this finding supports the contention that the use of this TNF inhibitor is strongly associated with the reactivation of latent tuberculosis into secondary tuberculosis. The tuberculosis risk ratio for adalimumab, which is a humanized antibody against TNF-α, was reported to be even higher at 19.9 (95% CI, 16.2-24.8) fold according to the data for a Spanish population [6, 29].

A similar number of people have been exposed to etanercept, and only nine cases of tuberculosis were reported at that time (p < 0.05). Using data collected from the adverse event reporting system of the FDA, Wallis found an increase in other granulomatous infections (histoplasma and fungus) associated with infliximab compared with etanercept (54 versus 32 cases per 100,000 patients treated; p < 0.001) [4]. The risk for TB with etanercept use appears to be less than for infliximab and adalimumab but is still present.

### Onset of TB in patients treated with anti-TNF-α medications-immunologic mechanisms

As discussed previously, after TB enters the lungs of a human host, most TB bacilli are ingested by alveolar macrophages and DCs, and the tuberculosis bacilli are isolated to the granuloma. Within the granuloma, accumulated monocytes differentiate into epithelioid cells and/or fuse to form giant cells. In the process, the presence of TNF-α is essential to a giant cell (Langhans-like cell) formation and maintenance (Figure 4, [26]), as it organizes granuloma in human body and functions in surveillance [30, 31]. The activation of focal adhesion kinases are linked to cell assembling and the formation of Langhans-like multinucleated giant cells [26].

In several clinical and experimental studies, when the containment of the bacteria in granulomas is obstructed by TNF-α neutralizing inhibitors (the biologics) previously latent TB bacilli may cause a disseminated TB disease [3, 4, 30, 31]. TNF signaling via TNF receptor-1 (TNF-R1) appears to be particularly required for host resistance to mycobacterial infection [32–34]. In a comparison of receptor knockout (KO) mice infected with Mycobacterium bovis, it was found that, while the bacterial burden of TNF-R1-deficient mice was significantly increased and the mice succumbed to mycobacterial infection, TNF-R2 KO mice were less sensitive [35]. TNF-R1 KO mice were associated with severe impairment in forming granulomas, with reduced macrophage recruitment and activation and diminished expression of adhesion molecules [36, 37]. Thus, anti-mycobacterial immunity is largely dependent on TNF signaling via the TNF-R1, while the activation of TNF-R2 appears to play a minor role [4, 35].

Etanercept is a diametric fusion protein consisting of two extracellular domains of the p55 TNF receptor (TNF-R1) and p75 TNF receptor (TNF-R2). The proinflammatory reaction through TNF-R2 is completely inhibited with etanercept, compared to that through TNF-R1, which is less inhibited. This may explain the relative decreased risk of TB in patients treated with etanercept compared to patients treated with infliximab or adalimumab.

In the human body, for the sterilization of TB bacillus in macrophages, the superoxide radical (peroxynitrite) production and the killing protein are also important; the sterilization is activated by IFN-γ in monocytes and macrophages [4, 31]. IFN-γ is capable of inducing CTL activity to mycobacterium-infected cells.

Experiments of nature have greatly elucidated the pathophysiology of immune disorders and susceptibility to TB. Chronic granulomatous disease, a congenital immunodeficiency, is associated with a lack of superoxide anion production in phagocytes and patients with this disorder are highly susceptible to mycobacteria. Mendelian susceptibility to mycobacterial infection disease (MSMD) is caused by the inherited deficiency of IFN –γ and IL-12-induced signal transduction [38, 39]. In addition, as we have discussed, anti-TNF-α therapy induces disseminated tuberculosis, obstructing granuloma formation. We can now conclude that the cytokines TNF-α and IFN-γ and superoxide anion production are essential to immunity against Mycobacterium tuberculosis.

### Anti-TNF-α inhibitors in Kawasaki disease

Infliximab, which is a neutralizing TNF inhibitor, has recently attracted attention as a therapy for Kawasaki disease (KD). This disease is at times severe and intractable, even with IVIG therapy. Infliximab significantly shortens fever duration and may obstruct coronary lump formation in KD [40, 41]. In such cases, excessive screening for latent tuberculosis infection and hesitation in the treatment may be ill-advised. Because a granuloma lesion (latent infection) of tuberculosis is overall less common in early childhood, the treatment with infliximab should be quickly administered in children with Kawasaki disease, when other treatments fail. From our literature review, we believe that this short-term usage of TNF inhibitors for will have little effect on the immunity against primary tuberculosis.

### Vaccines and anti-TNF-α medications

In 1998, the United States Centers for Disease Control announced that novel vaccines should be used in place of Bacille de Calmette et Guérin (BCG; M. bovis) for protection against tuberculosis worldwide. Yet BCG still remains in use throughout the world. Although the protective efficacy is well established against TB infection and its progression to clinical diseases during childhood [42], persistency of protective efficacy (cytotoxic CD8+ T cell activity) against *M. tuberculosis* is not fully provided with BCG vaccine outside the first decades of life. The development of a more effective vaccine against *M. tuberculosis* in the near future is highly desirable not only for the prevention of early progression of primary tuberculosis but also for the decline of patients infected with disseminated tuberculosis induced by aging and/or TNF-α inhibitors.

## Conclusions

The development of biologics is revolutionizing the treatment of immune-mediated inflammatory diseases such as RA, inflammatory bowel disease and juvenile idiopathic arthritis. On the other hand, this interception of inflammatory cytokines such as TNF-α, which are direct factors in the creation of inflammation, may restrain protective physiological reactions and cause reactions that can revive infectious diseases such as TB that are in dormancy. Rheumatologists, gastroenterologists, and other care providers prescribing biologics should understand these complications well and be familiar with the current knowledge of the roles of TNF- α in the human host as well as the pathophysiologic mechanisms of the TNF-α blockers in the development of secondary (disseminated) tuberculosis. Clinicians should be vigilant for TB and granulomatous infections when prescribing TNF inhibitors and other biologics and manage these side effects as a crucial component of the disease treatment.

## Abbreviations

TB: Tuberculosis
IFN: Interferon
TNF: Tumor necrosis factor
CTL: Cytotoxic T lymphocytes
MΦ: Macrophage
IFN: Interferon
IL: Interleukin
Th: T helper cell
GM-CSF: Granulocyte/macrophage colony-stimulating factor
IL-4: Interleukin-4
HB: Hepatitis type B
HC: Hepatitis type C.
